# Sensitivity Improvement of Extremely Low Light Scenes with RGB-NIR Multispectral Filter Array Sensor

**DOI:** 10.3390/s19051256

**Published:** 2019-03-12

**Authors:** Seunghoon Jee, Moon Gi Kang

**Affiliations:** Department of Electrical and Electronic Engineering, Yonsei University, 50 Yonsei Road, Seodaemun-gu, Seoul 03722, Korea; jeedang1109@hanmail.net

**Keywords:** extremely low light, sensitivity improvement, near infrared, multispectral filter array

## Abstract

Recently, several red-green-blue near-infrared (RGB-NIR) multispectral filter arrays (MFAs), which include near infrared (NIR) pixels, have been proposed. For extremely low light scenes, the RGB-NIR MFA sensor has been extended to receive NIR light, by adding NIR pixels to supplement for the insufficient visible band light energy. However, the resolution reconstruction of the RGB-NIR MFA, using demosaicing and color restoration methods, is based on the correlation between the NIR pixels and the pixels of other colors; this does not improve the RGB channel sensitivity with respect to the NIR channel sensitivity. In this paper, we propose a color restored image post-processing method to improve the sensitivity and resolution of an RGB-NIR MFA. Although several linear regression based color channel reconstruction methods have taken advantage of the high sensitivity NIR channel, it is difficult to accurately estimate the linear coefficients because of the high level of noise in the color channels under extremely low light conditions. The proposed method solves this problem in three steps: guided filtering, based on the linear similarity between the NIR and color channels, edge preserving smoothing to improve the accuracy of linear coefficient estimation, and residual compensation for lost spatial resolution information. The results show that the proposed method is effective, while maintaining the NIR pixel resolution characteristics, and improving the sensitivity in terms of the signal-to-noise ratio by approximately 13 dB.

## 1. Introduction

Recently, several attempts have been made to utilize near infrared (NIR) band information. Multispectral images observed in various spectrum bands, including both visible and NIR bands, have been used in remote sensing applications [1,2]. As each spectral band provides a different type of information, the spectral bands were selectively used in observing the multi-spectral images. In surveillance [3] and night vision cameras [4], the NIR band is particularly useful under low lighting or invisible NIR lighting conditions. The NIR band is also used in biometric [5], face matching [6], and face recognition [7] applications, which have been studied based on the intrinsic reflectivity of the skin or eyes under NIR illumination. As the NIR reflection is material dependent, it is also used in material classification [8] and illuminant estimation [9]. NIR images can be used in image enhancement applications such as image dehazing [10].

**Figure 1:**
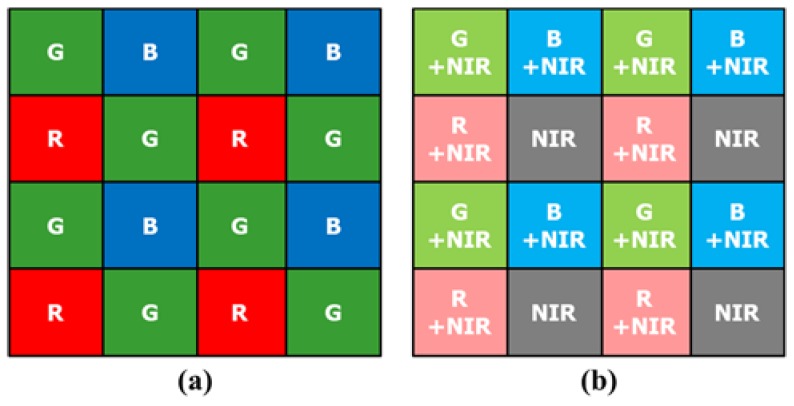
(**a**) Bayer color filter array [11]; (**b**) RGB-NIR multispectral filter array [27].

Most digital imaging devices use a color filter array (CFA) to reduce equipment cost and size instead of using three sensors and optical beam splitters. The Bayer CFA, comprising the primary colors red, green, and blue (R, G, and B), is a widely used CFA [11]. Figure 1a shows the Bayer CFA. Recently, methods using a new CFA have been studied to overcome the limited sensitivity of the Bayer CFA under extremely low light conditions. This is because the amount of absorbed light decreases on account of the RGB color filters. Thus, sensitivity improvement methods utilizing an invisible light source have been proposed in recent years [12,13,14,15,16,17,18,19]. Because the wavelength of an invisible light source is different from that of visible light, the invisible and visible light images can be captured simultaneously. New CFA patterns [20] and demosaicing methods [21,22,23,24], containing NIR pixels in the pattern have been proposed. Figure 1b shows an example of a red-green-blue NIR (RGB-NIR) multispectral filter array (MFA). Bennett et al. [12] constructed an imaging system that can simultaneously capture color and NIR images. For a pair of color and NIR images, the proposed system outperformed the reconstruction methods reported in [25,26]. Yan et al. [16] preserved the necessary details and edges of a color image by considering the structural differences between the NIR and color images. These studies reported good results for enhancing color images of low light scenes. However, existing methods have an implied restriction, regarding how dark the environment can be. If the lighting conditions are darker than that assumed in these methods, they will not work well.

**Figure 2:**
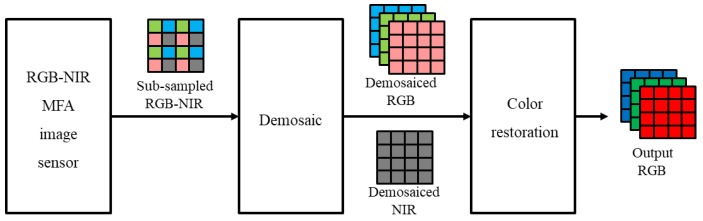
RGB-NIR multispectral filter array system framework.

**Figure 3:**
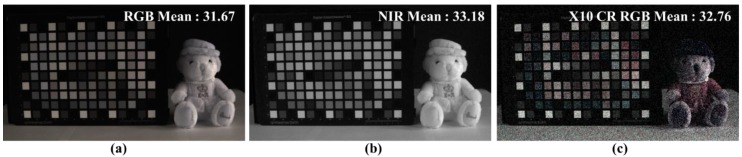
Color restored results [27] under extremely low light condition (0.01lx, incandescent); (**a**) input RGB image; (**b**) input NIR image; (**c**) 10 times gained image from color restored RGB.

Generally, to utilize the RGB-NIR MFA, two steps are involved (Figure 2): Demosaicing and color restoration [20,27]. The demosaicing process, also called color interpolation, is a process of converting sub-sampled R, G, B, and NIR channels into full resolution images. Figure 3 shows an image taken under extremely low (0.01lx) incandescent light conditions. From Figure 3a,b, it is possible to obtain high sensitivity color images from the RGB-NIR MFA by incandescent lighting, with a large amount of NIR energy under extremely low light conditions. However, demosaiced RGB channels contain the NIR band, as shown in Figure 3a, which is different from typical RGB channel color reproduction. Therefore, a color restoration process is required to estimate and remove the included NIR band. Figure 3c shows the results of the color restoration algorithm for the image, shown in Figure 3a [27], with the brightness similar to that of the NIR channel, a gain of 10 times. The two processes, using the NIR channel from the images (Figure 3), indicate that the sensitivity of the color image is not improved with respect to the sensitivity of the NIR channel under extremely low light conditions.

Various post-processing algorithms have been studied using the NIR band for this problem. For example, image fusion methods [28] fuse the NIR and RGB channels. Fusion methods focus on identifying invisible or indistinguishable subjects in the visible band, while maintaining the RGB color components. Although object discrimination ability improved, the sensitivity remains poor. Therefore, the local contrast and color are distorted. Another technique involves using a guided filtering (GF) method [29,30] that considers the linear correlation between the luminance (Y) channel of the visible band, and the NIR channel, to reflect the sensitivity characteristics of the NIR channel. This method uses linear regression between the Y and NIR channels to estimate the linear coefficients. The NIR channel is reconstructed into Y channels by using the coefficients. This method is also used for reconstructing low illuminance images from a flashing image. However, under extremely low light conditions, a visible image with a high level of noise makes it difficult to estimate the linear coefficients accurately. Moreover, the resolution of the Y channel, reconstructed from the linear coefficients, is not up to that of the NIR channel. To solve this problem, five cost functions, and an algorithm to minimize them, have been proposed [31]. This algorithm is more stable than the above algorithms under extremely low light conditions; however, it is difficult to realize NIR channel level sensitivity because of the trade-off between the five factors in the cost minimization process.

In this paper, we propose a post-processing method that can enhance the spatial resolution, while improving the brightness and signal-to-noise ratio (SNR) of the RGB channels, by reflecting the sensitivity of the NIR channel. High sensitivity NIR channels, at very low light levels, have better spatial resolution and sensitivity than noisy RGB channels. On the other hand, RGB channels contain color information and local contrasts that represent the colors. Hence, we propose a method to improve the resolution and SNR of the R, G, and B channels by reconstructing the NIR channel. In particular, we propose a smoother method to improve the accuracy of linear coefficients of guided filtering in extremely low light conditions. In addition, we propose residual information compensation to recover spatial resolution information and texture, lost in the guided filtering process.

This paper proposes an edge preserving smoother based pre-processing algorithm to estimate the linear coefficients of the guided filter accurately. The edge preserving smoother proposed in [16] estimates the kernel from the sensitive NIR channel and applies the kernel to the Y channel. The smoothened Y channel removes the noise and texture components, that interfere with linear coefficient estimation, along the strong edges of the NIR channel. This procedure allows the guided filter to estimate the correct coefficient in extremely low light scenes. In addition, this paper proposes a post-processing algorithm to improve the resulting resolution of the guided filter. Guided filtering is used to estimate the residual component from the NIR channel and compensate for the insufficient spatial resolution of the reconstructed Y channel. The residual component is the spatial resolution information of the missing NIR channel in the guided filtering process. Finally, the proposed algorithm maintains the color and local contrast of the RGB channel, through guided filtering and outputs the result with NIR channel level sensitivity and spatial resolution.

The remainder of this paper is organized as follows. In Section 2, the research problem is detailed. In Section 3, the proposed post-processing method based on guided filtering is described. In Section 4, we present the results of conducted experiments, and compare the results of the proposed method with those of other post-processing algorithms. Our conclusions are presented in Section 5.

## 2. Problem Statement

**Figure 4:**
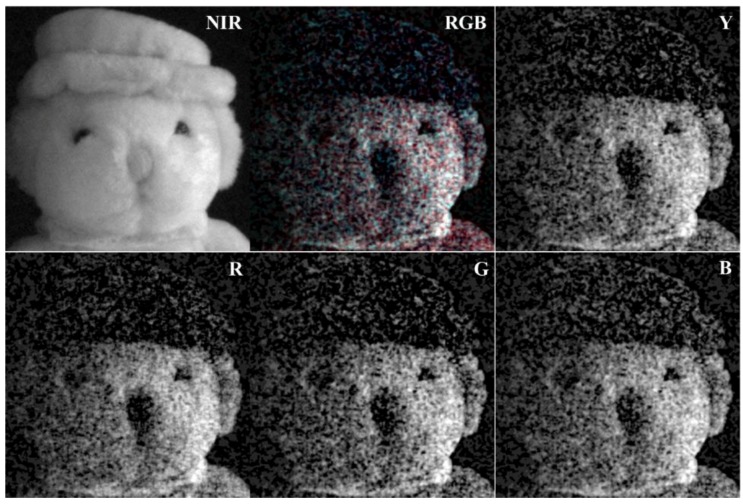
Color restoration results by channel [27] under extremely low light condition (0.01lx, incandescent).

The color restoration results of the RGB-NIR MFA under extremely low light conditions include a high level of spatial and color noise, as shown in Figure 3c. To analyze the characteristics of the noise, Figure 4 shows the color restoration results with respect to the type of channel. The RGB channels show a similar level of noise, whereas the noise level in the Y channel is more than that in the NIR channel. On the other hand, the NIR channel is a high sensitivity image, with low noise and accurate texture information of the subject. However, it does not have the local brightness or contrast shown in the visible band. This is because the hat and the head of the subject are not clearly distinguished.

In short, the rationale of the proposed post-processing method is that the reconstructed image has NIR channel level brightness and sensitivity. Moreover, the color image has an NIR channel level SNR and color reproduction at the color restored RGB result level. Table 1 lists the characteristics of the color restored RGB result image, obtained using the proposed post-processing algorithm. The brightness, sensitivity, noise, and spatial resolution characteristics of the image should follow those of the NIR channel. On the other hand, the hue, saturation, and local contrast must follow those of the color restored RGB channel to maintain color reproduction.

The proposed post-processing algorithm follows the guided filtering method [29]. The guided filtering estimates the linear coefficients between the NIR channel and the Y channel by using a linear regression method. From the estimated linear coefficients, the NIR channel is reconstructed into associations with local brightness and contrast similar to the Y channel. In addition, a smoothing process, such as mean operation, for the linear coefficients can help reduce the influence of noise. However, it is difficult to estimate the linear coefficients accurately because the noise under extremely low light conditions occurs, not only in the high frequency band, but also in the low frequency band. The noise generated up to the low frequency band can be removed using a strong smoothing filter. However, this will damage both the occlusions of the subject and the strong edge areas, thus reducing the accuracy of linear coefficient estimation in the strong edge regions and generating artifacts in the resulting image. 

**Figure 5:**
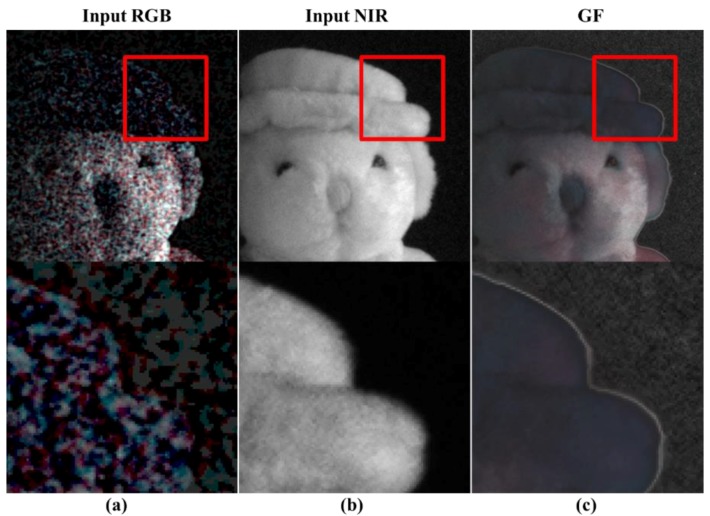
Guided filtering [29] results under extremely low light condition (0.01lx, incandescent); (**a**) input RGB image; (**b**) input NIR image; (**c**) the post-processing result by guided filtering (GF).

Figure 5 shows the input channels, RGB channels, NIR channel, and guided filtering results. Figure 5c shows the artifacts due to the error in estimating the linear coefficients during smoothing. In the background region, noise characteristics, amplified over the NIR channel, can be seen. This is due to the failure in accurately estimating the linear coefficients in the noise region, as shown in Figure 5a. In addition, the guided filtering result shows that there is no texture information on the hat. Therefore, the proposed algorithm performs guided filtering correction, thus establishing a post-processing algorithm suitable for extremely low light conditions.

## 3. Proposed Post-Processing Method Based on Guided Filtering

### 3.1. Post-Processing Framework

**Figure 6:**
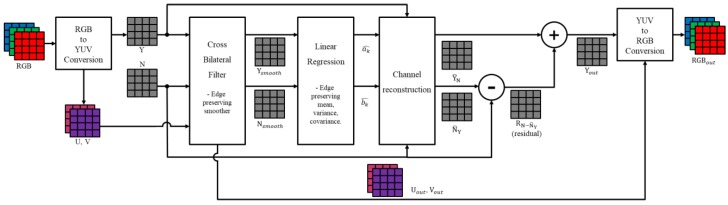
The framework of proposed post-processing.

To solve the problem of noise, linear coefficients, and texture information of the NIR channel, under extremely low light conditions, this paper proposed a post-processing method based on guided filtering. The proposed algorithm comprises three steps: edge preserving smoothing, guided filtering, and residual compensation. Figure 6 shows the framework of the proposed post-processing algorithm.

First, the color restored RGB channel, converted into the YUV domain, is divided into luminance and chrominance channels, and the Y channel is inputted to the edge preserving smoother along with the NIR channel. The reason for converting to the YUV domain is to separate color components of RGB, such as saturation and hue, to prevent color change during the algorithm execution. The U and V channels, except for the luminance component, contain color components and represent color information and color noise.

The edge preserving smoother can accurately preserve the strong edges and occlusions of the object by estimating the edge from the sensitive NIR channel. The smoothing kernel, estimated from the NIR channel, has little influence on the Y channel in terms of the noise. Thus, it can remove the low frequency noise and preserve the strong edges. In addition, the color noise, existing in the U and V channels, is removed through the kernel estimated from the NIR channel, thus maintaining the correct color at the boundary of the object. In this paper, a cross bilateral filter (CBF) [26] was used as an edge preserving smoother. The NIR channel passing through the smoothing filter contains local brightness and local contrast information, and the noise and texture information can be removed. This can improve the regional distribution similarity between the Y and NIR channels in estimating the linear coefficients. Moreover, the smoothened Y and NIR channels maintain a strong edge, and the guided filter can accurately estimate the linear coefficient of change at the boundary of the object.

Guided filtering estimates the linear coefficients through linear regression. The estimated linear coefficients help reconstruct the NIR channel to create a new Y channel with improved sensitivity. The proposed algorithm smoothens the estimated linear coefficients by using the CBF kernel estimated from the NIR channel. This stabilizes the small errors that occur in the estimation process and preserves the linear coefficients in the occlusion of the object. In addition, the proposed algorithm updates the reconstructed Y channel, by estimating the texture and high frequency components (the residual information) that have not yet been compensated via guided filtering.

Finally, the Y channel, reconstructed from the NIR channel, has a local contrast of the input Y channel and local brightness characteristics. In addition, the reconstructed Y channel has NIR level sensitivity, noise, and spatial resolution characteristics. Similarly, the smoothened U and V channels are suppressed from noise in all the frequency bands, and they can maintain local color characteristics.

### 3.2. Edge Preserving Smoother and Linear Coefficients

The key assumption in the guided filtering is the local linear model between the NIR and Y channels. The local linear model can be expressed as follows:

(1) $$Y_{S}=a_{S}N_{S}+b_{S},$$

where S is the local area of the object in an image, Y and N are the Y, and NIR channels, respectively, and $a_{S}$ and $b_{S}$ are the linear coefficients. Equation (1) can be expressed as follows for the current pixel position:

(2) $$I_{Y}\left(p\right)=a\left(p\right)I_{N}\left(p\right)+b\left(p\right),\;\forall p\in S,$$

where $I_{Y}\left(p\right)$ and $I_{N}\left(p\right)$ are the Y and NIR channel pixel values at the p**^th^** position, respectively. The linear coefficients are assumed to be constant in S. To obtain the linear coefficients using a simple linear regression model, the following equations have been proposed [32]:

(3) $$a_{S}=\frac{Cov\left(N_{S},Y_{S}\right)}{Var\left(N_{S}\right)+\varepsilon }\;,$$

(4) $$b_{S}=Mean\left(Y_{S}\right)-a_{S}Mean\left(N_{S}\right),$$

where ε is a small constant to prevent division by zero, $Cov\left(N_{S},Y_{S}\right)$ is the covariance between the NIR and Y channels in the region S, $Var\left(N_{S}\right)$ is the variance of NIR in the region S, and $Mean\left(Y_{S}\right)$ is the mean value of the Y channel in the region S. The following equations can be used to obtain the mean, variance, and covariance from the NIR and Y channels:

(5) $$Mean_{S}\left(N\left(p\right)\right)=\underset{p,q\in S}{\sum }I_{N}\left(q\right)/\left|S\right|,$$

(6) $$Var_{S}\left(N\left(p\right)\right)=\underset{p,q\in S}{\sum }{\left(I_{N}\left(q\right)\right)}^{2}/\left|S\right|-{\left(\underset{p,q\in S}{\sum }I_{N}\left(q\right)/\left|S\right|\right)}^{2},$$

(7) $$Cov_{S}\left(N\left(p\right),Y\left(p\right)\right)=\underset{p,q\in S}{\sum }I_{N}\left(q\right)\;I_{Y}\left(q\right)/\left|S\right|-Mean\left(N\left(p\right)\right)\cdot Mean\left(Y\left(p\right)\right),$$

where q is the pixel position in the region S, |S| is the number of pixels in the region S, and Mean(N(p)) is the mean value at the current pixel position p in the NIR channel. Var(N(p)) is the variance value at the current pixel position p in the NIR channel, and Cov(N(p),Y(p)) is the covariance value at the current pixel position p between the Y and NIR channels.

The linear coefficients of the guided filter, mentioned in Section 2, show errors because the covariance is estimated from the noisy Y channel to the NIR channel. In other words, as the noise level increases, the linearity of the Y channel with the NIR channel decreases, and the accuracy of the coefficient $a_{S}$ decreases. An inaccurately predicted $a_{S}$ value can lead to an inaccurate prediction of $b_{S}$ in Equation (4). In conclusion, it is necessary to improve the accuracy of $Cov\left(N_{S},Y_{S}\right)$ correlated with the Y channel.

**Figure 7:**
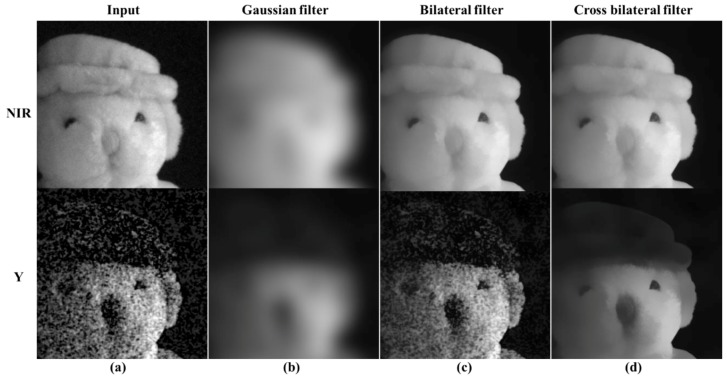
The comparison of various smoother results; (**a**) input channels; (**b**) Gaussian filtering results; (**c**) bilateral filtering results; (**d**) cross bilateral filtering results.

A Gaussian filter is incorporated in the guided filtering method [29] to improve the accuracy of linear coefficient estimation. However, when the noise level is high, the strong edges are blurred. Therefore, this paper proposes an edge preserving smoother by pre-processing the guided filter. Figure 7 compares the various Gaussian function based smoothing filter results for the NIR and Y channels. The filter characteristics are as follows: Gaussian filter with no edge preservation, a bilateral filter with edge preserving, and a cross bilateral filter to estimate the kernel from the guide image. As described above, the Gaussian filter result (Figure 7b) shows that although the influence of the noise is reduced, the occlusion of the object is significantly blurred. In the bilateral filter results, shown in Figure 7c, the noise removal of the Y channel is limited. This is the limitation of the kernel estimated from the Y channel, which contains significant noise under extremely low light conditions, even for the edge preserving smoother. The cross bilateral filter results (Figure 7d) show that the noise is effectively removed from the Y channel, and the strong edge and occlusion regions are preserved in both the NIR and Y channels. Moreover, the texture information is effectively removed from the NIR channel. Hence, we used the CBF as a pre-processing step in the guide filtering to take advantage of the highly sensitive NIR channel characteristics; the CBF replaces the mean operation. The existing mean operation is a uniform blur method and is replaced with an edge preserving smoother. The CBF can be expressed as follows:

(8) $$Mean_{N,S}^{CBF}\left(Y\left(p\right)\right)=\frac{1}{W_{N,S}^{CBF}\left(p\right)}\underset{p,q\in S}{\sum }G_{\sigma_{s}}\left(\left|\left|p-q\right|\right|\right)G_{\sigma_{r}}\left(\left|I_{N}\left(p\right)-I_{N}\left(q\right)\right|\right)I_{Y}\left(q\right),$$

(9) $$W_{N,S}^{CBF}\left(p\right)=\underset{p,q\in S}{\sum }G_{\sigma_{s}}\left(\left|\left|p-q\right|\right|\right)G_{\sigma_{r}}\left(\left|I_{N}\left(p\right)-I_{N}\left(q\right)\right|\right),$$

where $Mean_{N,S}^{CBF}\left(Y\left(p\right)\right)$ is the CBF result of the Y channel at the p^th^ pixel, obtained by applying a bilateral filter kernel estimated from the NIR channel, $G_{\sigma_{s}}$ and $G_{\sigma_{r}}$ are the Gaussian functions for the spatial and range kernels, respectively, $\sigma_{s}$ and $\sigma_{r}$ are the smoothing parameters, and $W_{N,S}^{CBF}\left(p\right)$ is the normalized factor at the p^th^ pixel. For smoother results in Figure 7, for a 48 (16 + 16 + 16) bit color input image, the parameter settings for filters are Gaussian filter σ = 300, bilateral filter $\sigma_{s}$ = 300, bilateral filter $\sigma_{r}$ = 5000, cross bilateral filter $\sigma_{s}$ = 300, and bilateral filter $\sigma_{r}$ = 5000. The filter kernel size (S) is 31 × 31. The variance and covariance values estimated using the proposed CBF are as follows:

(10) $$Var_{N,S}^{CBF}\left(N\left(p\right)\right)=\frac{1}{W_{N,S,Var}^{CBF}\left(p\right)}\underset{p,q\in S}{\sum }{\left(G_{\sigma_{s}}\left(\left|\left|p-q\right|\right|\right)G_{\sigma_{r}}\left(\left|I_{N}\left(p\right)-I_{N}\left(q\right)\right|\right)I_{N}\left(q\right)\right)}^{2}\;-Mean_{N}^{CBF}{\left(N\left(p\right)\right)}^{2},$$

(11) $$W_{N,S,Var}^{CBF}\left(p\right)=\underset{p,q\in S}{\sum }G_{\sigma_{s}}{\left(\left|\left|p-q\right|\right|\right)}^{2}G_{\sigma_{r}}{\left(\left|I_{N}\left(p\right)-I_{N}\left(q\right)\right|\right)}^{2},$$

(12) $$Cov_{N,S}^{CBF}\left(N\left(p\right),Y\left(p\right)\right)=\frac{1}{W_{N,S,Var}^{CBF}\left(p\right)}\underset{p,q\in S}{\sum }G_{\sigma_{s}}{\left(\left|\left|p-q\right|\right|\right)}^{2}G_{\sigma_{r}}{\left(\left|I_{N}\left(p\right)-I_{N}\left(q\right)\right|\right)}^{2}I_{N}\left(q\right)I_{Y}\left(q\right)\;-Mean_{N}^{CBF}\left(N\left(p\right)\right)\cdot Mean_{N}^{CBF}\left(Y\left(p\right)\right),$$

where $Var_{N,S}^{CBF}\left(N\left(p\right)\right)$ is the variance value of the smoothing filtered NIR channel at the p^th^ pixel, obtained by applying a bilateral filter kernel estimated from the NIR channel, $Cov_{N,S}^{CBF}\left(N\left(p\right),Y\left(p\right)\right)$ is the variance value of the smoothing filtered Y channel at the p^th^ pixel, obtained by applying a bilateral filter kernel estimated from the NIR channel, and $W_{N,S,Var}^{CBF}\left(p\right)$ is the normalized factor for the variance at the p^th^ pixel. For conditions similar to the smoothed Y channels with texture information and noise removed, the NIR channel also needs to pass through the same bilateral filter kernel. This process minimizes the effect of texture on the covariance estimate and improves the accuracy of estimating the correlation between the local brightness of the objects on each channel. The linear coefficients $a_{s}^{CBF}$ and $b_{s}^{CBF}$ associated with the CBF can be expressed as follows:

(13) $$a_{S}^{CBF}=\frac{Cov_{N,S}^{CBF}\left(N\left(p\right),Y\left(p\right)\right)}{Var_{N,S}^{CBF}\left(N\left(p\right)\right)+\varepsilon },$$

(14) $$b_{S}^{CBF}=Mean_{N,S}^{CBF}\left(Y\right)-a_{S}Mean_{N,S}^{CBF}\left(N\right).$$

The linear coefficients estimated from the smoothed image, are more accurate than the original values, but pass through the estimated bilateral filter kernel from the NIR channel to eliminate some possible estimation errors. The linear coefficients passing through the CBF kernel are expressed as follows:

(15) $${\hat{a}}_{S}\left(p\right)=Mean_{N,S}^{CBF}\left(a_{S}\left(p\right)\right),$$

(16) $${\hat{b}}_{S}\left(p\right)=Mean_{N,S}^{CBF}\left(b_{S}\left(p\right)\right),$$

(17) $$I_{{\hat{Y}}_{N}}\left(p\right)=\hat{a}\left(p\right)I_{N}\left(p\right)+\hat{b}\left(p\right),$$

where ${\hat{a}}_{S}$ and ${\hat{b}}_{S}$ are the smoothed linear coefficients, ${\hat{Y}}_{N}$ is the Y channel reconstructed from the NIR channel by using the linear coefficients ${\hat{a}}_{S}$ and ${\hat{b}}_{S}$.

**Figure 8:**
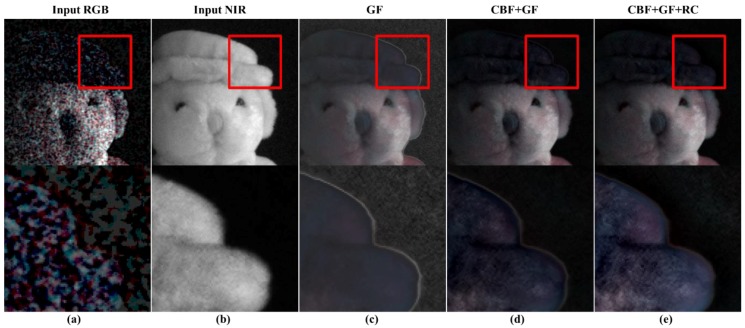
Comparison of step-by-step results of the proposed post-processing; (**a**) input RGB; (**b**) input NIR; (**c**) guided filter (GF) result; (**d**) the result of using cross bilateral filter (CBF) as the post processing of guided filtering; (**e**) compensation of residual (RC) information to (**d**).

**Figure 9:**
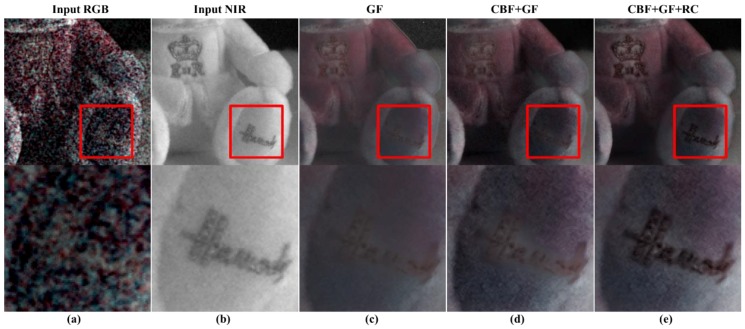
Comparison of step-by-step results of the proposed post-processing; (**a**) input RGB; (**b**) input NIR; (**c**) guided filter (GF) result; (**d**) the result of using cross bilateral filter (CBF) as the post processing of guided filtering; (**e**) compensation of residual (RC) information to (**d**).

**Figure 10:**
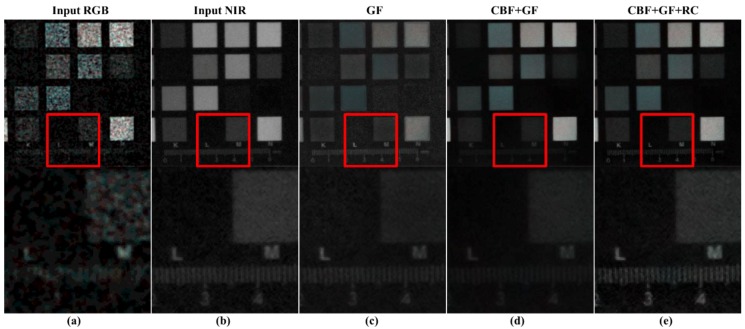
Comparison of step-by-step results of the proposed post-processing; (**a**) input RGB; (**b**) input NIR; (**c**) guided filter (GF) result; (**d**) the result of using cross bilateral filter (CBF) as the post processing of guided filtering; (**e**) compensation of residual (RC) information to (**d**).

Figure 8c,d show the guided filtering results, and proposed CBF + guided filtering results, respectively. The accuracy of linear coefficient estimation is improved with the use of CBF, showing stable results in the background. Figure 8d shows that the texture components on the cap are more effectively restored, compared to the guided filtering results. However, Figure 9d shows that the texture on the foot of the doll is not well restored. Figure 10d shows that the texture is not well reconstructed in the resolution chart area at the bottom of the color chart. To solve this problem, we perform post-processing of the guided filter.

### 3.3. Compensation with Residual Information of NIR

The texture information, in the channel reconstruction results of the guided filter, is lacking. Because of the area wise computation of the linear coefficients, it is difficult to reconstruct the texture. To obtain spatial resolution at the NIR channel level, this paper proposes a residual information compensation process. The residual information is defined as the information lost in the NIR channel among the textures resulting from guided filtering. To estimate the residual information, the NIR channel is reconstructed using the Y channel. This process traces the loss process through the channel other than the NIR channel to estimate the texture information lost during guided filtering. The process of reconstructing the NIR channel, by using the linear coefficients estimated in the previous sub section is as follows:

(18) $$I_{{\hat{N}}_{Y}}\left(p\right)=\frac{I_{Y}\left(p\right)-\hat{b}\left(p\right)}{\hat{a}\left(p\right)+\varepsilon },$$

where ${\hat{N}}_{Y}$ is the NIR channel reconstructed from the Y channel. Equation (18) is a transformed version of Equation (17). The residual information is estimated as follows:

(19) $$R_{N-{\hat{N}}_{Y}}=N-{\hat{N}}_{Y},$$

where $R_{N-{\hat{N}}_{Y}}$ is the residual information and the difference between the NIR channel and the channel ${\hat{N}}_{Y}$ reconstructed from the Y channel. The process of compensating for the residual information is as follows:

(20) $$Y_{out}={\hat{N}}_{N}+R_{N-{\hat{N}}_{Y}},$$

where $Y_{out}$ is the proposed result of the luminance channel. The results of the chrominance channels are expressed as:

(21) $$U_{out}=Mean_{N,S}^{CBF}\left(U\right),$$

(22) $$V_{out}=Mean_{N,S}^{CBF}\left(V\right),$$

where $U_{out}$ and $V_{out}$ are the resulting chrominance channels of the proposed algorithm, and U and V are the chrominance channels of the input image.

Figure 8e, Figure 9e, and Figure 10e show the GF + CBF results in compensating for the residual (residual compensation, RC) information. Figure 8e shows that the white line artifact, shown in Figure 8d, is suppressed, and the texture becomes richer. Figure 9e and Figure 10e show that the missing texture information is well compensated from Figure 9d and Figure 10d.

## 4. Experimental Results

To verify the performance of the proposed algorithm under extremely low light conditions, we used directly captured images. The image, shown in Figure 3a, was taken in 0.01 lx incandescent light under extremely low light conditions. We measured the R, G, B, and NIR channels with full resolution by using a multi-spectral filter wheel with a single sensor. The resolution of the image used in the experiment was 1600 × 1200, and it was photographed with 16 bits per pixel of one channel. By using the color restoration algorithm proposed in [27], we obtained the RGB image, shown in Figure 3c, with a gain of 10 times gain. The proposed post-processing uses the RGB color image (Figure 3c) and the NIR gray image (Figure 3b) as the input image.

In this section, we compare various conventional methods (CM) to verify the performance of the proposed algorithm. First, we compared the core process guided filtering (CM1) [29]. This algorithm is widely used as a sensitivity enhancement algorithm that utilizes the NIR channel under general illumination conditions rather than under extremely low light conditions. Second, it was compared with the image fusion method (CM2) [28]. This method helps improve the information amount by fusing the visible and invisible bands to facilitate the material classification using the NIR channel. Finally, it is compared with the cost minimization method (CM3) [31]. This method proposes five cost functions that compensate the sensitivity characteristics of the NIR channel while maintaining the color characteristics of the RGB channel under extremely low light conditions and obtain the results through a minimization process. The experimental environment used to measure the computing time included C++ code on Intel i7-6700k CPU 4 Ghz, Windows 10, and Visual Studio 2015. The algorithm execution times for CM1, CM2, CM3, and the proposed method are 0.880 s, 1.213 s, 4.520 s, and 1.107 s, respectively. The proposed method is faster than the other algorithms except CM1, which has fast computation time because it has guided filtering included in the proposed method.

**Figure 11:**
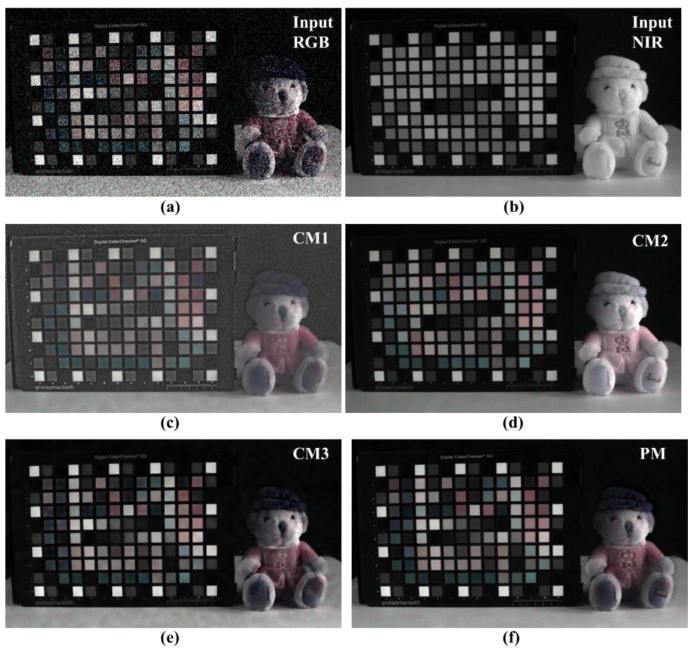
Comparison of various post- processing results (whole images); (**a**) input RGB; (**b**) input NIR; (**c**) conventional method 1 [29]; (**d**) conventional method 2 [28]; (**e**) conventional method 3 [31]; (**f**) proposed method.

Figure 11 shows the input image, results of the three conventional methods, and the full image obtained using the proposed algorithm. In Section 2, the results of CM1 obtained using guided filtering, were poor because the linear coefficients could not be accurately determined under extremely low light conditions. The results of CM2 show that the noise characteristics of the NIR are generally followed. However, based on the local contrast and brightness of the NIR, the color reproduction of the RGB channel can’t be followed. The result of CM3 follows the color reproduction of RGB; however, the low frequency noise that occurs under extremely low light conditions is not removed, and the noise characteristic of the NIR level is not exhibited. The proposed method (PM) results follow the RGB color reproduction and show NIR level noise level at the same time. Thus, the proposed algorithm shows better noise suppression, and color reproduction ability, than conventional methods.

**Figure 12:**
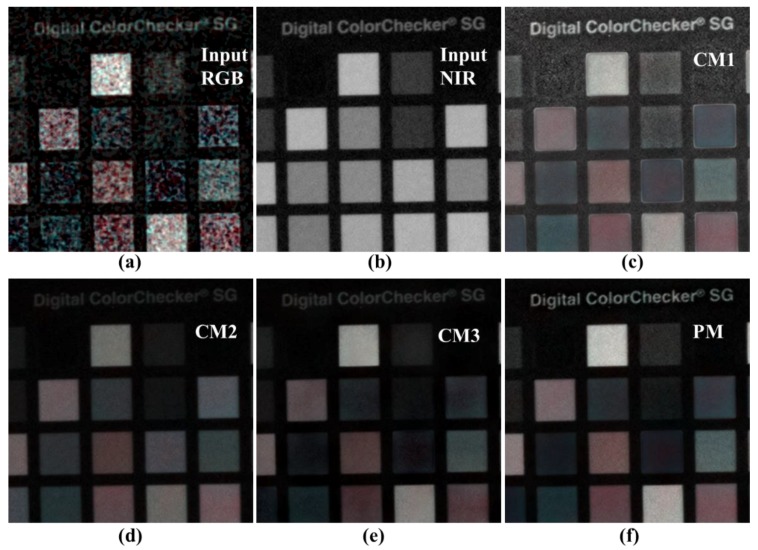
Comparison of various post-processing results (cropped images); (**a**) input RGB; (**b**) input NIR; (**c**) conventional method 1 [29]; (**d**) conventional method 2 [28]; (**e**) conventional method 3 [31]; (**f**) proposed method.

Figure 12 shows the image of the color chart and text area shown in Figure 11. First, the proposed algorithm for the writing area shows the expressive power of the NIR level, and no noise is amplified in the background. This was achieved by compensating for the residual information. In the color patch, only CM2 and PM are effectively expressed without the influence of noise. However, as CM2 follows the local brightness of the NIR, the color reproduction power falls short of the PM result. The good noise characteristics and color reproduction ability of the PM can be attributed to the proposed edge preserving smoother.

**Figure 13:**
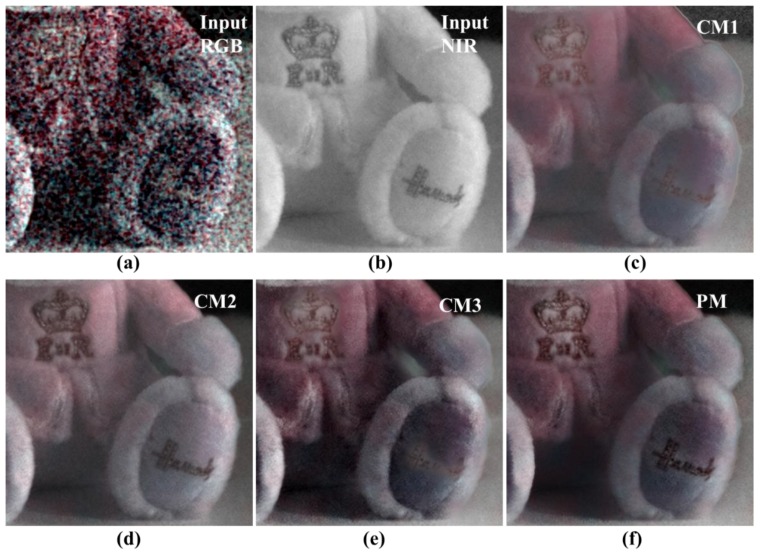
Comparison of various post- processing results (cropped images); (**a**) input RGB; (**b**) input NIR; (**c**) conventional method 1 [29]; (**d**) conventional method 2 [28]; (**e**) conventional method 3 [31]; (**f**) proposed method.

Figure 13 shows the image of the doll’s body area shown in Figure 11. The experimental results show that CM3 and PM effectively portray the RGB colors in terms of the color reproduction ability. However, the texture information regarding the doll’s body, and foot reflected by CM2 and PM, are not up to the mark. As shown in Figure 12, the edge preserving smoothing of the proposed algorithm helps improve the accuracy of estimating the linear coefficients via guided filtering, and the compensated residual information helps improve the texture information.

SNR [33] was used for the numerical comparison of sensitivity improvement in extremely low light conditions. The equation of SNR is expressed as:

(23) $$SNR_{dB}=20log_{10}\frac{\mu_{I}}{\sigma_{I}}\;,$$

where $SNR_{dB}$ represents the SNR, and the unit is decibels (dB) in the log scale. In addition, $\mu_{I}$ is the average of the measurement region in the image, and $\sigma_{I}$ denotes the standard deviation of the measurement region in the image.

**Figure 14:**
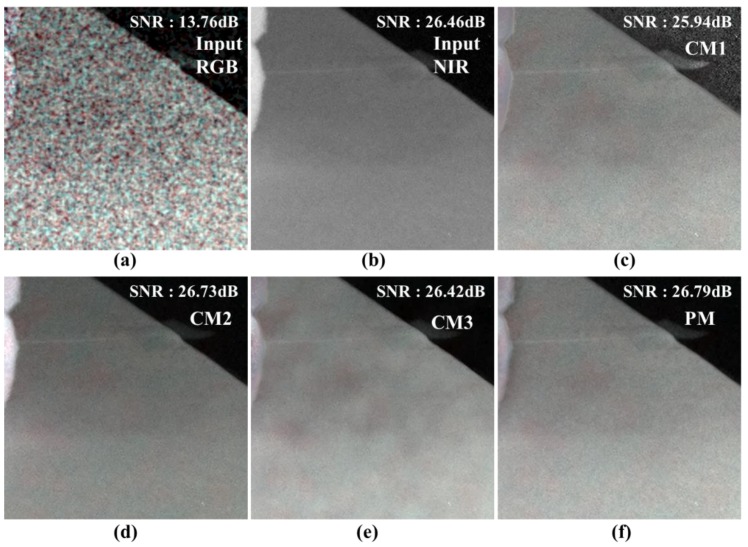
Comparison of various post- processing results (cropped images); (**a**) input RGB; (**b**) input NIR; (**c**) conventional method 1 [29]; (**d**) conventional method 2 [28]; (**e**) conventional method 3 [31]; (**f**) proposed method.

Figure 14 shows the white flat area and the SNR were measured in this area. The SNR values of the color images in Figure 14 represent average values measured on the R, G, and B channels, respectively. The NIR channel is a single SNR value. Figure 14 shows the best noise characteristics of PM and CM2. The fusion method results reflect the local brightness of the NIR, which improves the noise characteristics. The proposed algorithm follows the local brightness of RGB and conforms more closely to the development purpose of following the noise characteristic of NIR. Table 2 shows the results of Figure 14 in more detail. The average SNR of the input image is 13.76 dB, which means that the noise is considerably large. The CM1 result has a higher SNR than RGB but the lowest value among other comparison algorithms. CM2 has a 12.97 dB improvement over RGB and higher SNR than NIR. This is a result of faithfully reflecting the NIR information. The result of CM3 is lower than that of fusion but it is 12.66 dB better than that of RGB. It faithfully follows the local brightness of RGB, but it does not suppress low frequency noise. The proposed method shows the highest SNR and 13.03 dB improvement over RGB. This is higher than that of the fusion method, but the difference is insignificant. The high numerical value of the proposed algorithm shows that the sensitivity characteristic of NIR is reflected in the fusion level.

**Figure 15:**
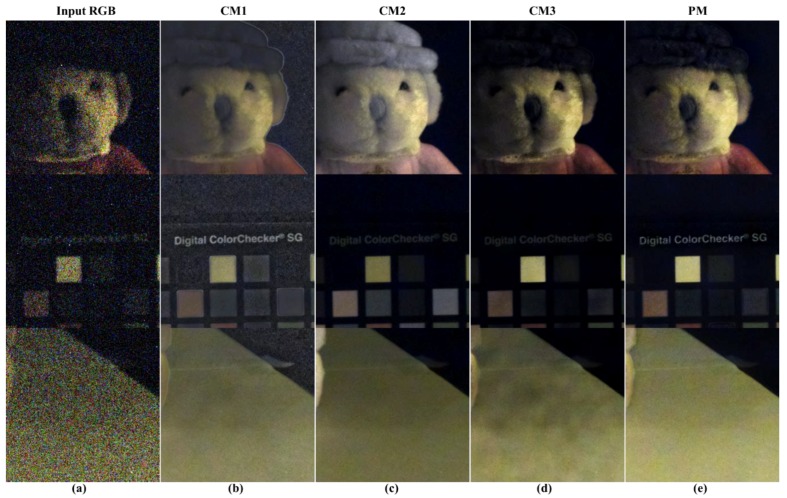
Comparison of various post- processing results (sodium lamp, 0.01lx, cropped images); (**a**) input RGB; (**b**) conventional method 1 [29]; (**c**) conventional method 2 [28]; (**d**) conventional method 3 [31]; (**e**) proposed method.

In addition to incandescent lamps, the same experiment was conducted in an extremely low illumination environment, including the NIR band such as a sodium lamp. This is to verify the proposed method in different lighting conditions (color temperature and lighting spectrum). Fluorescent lamps or LED lamps are excluded from the comparison because they cannot utilize the NIR band without artificially adding NIR bands. Figure 15 shows the result of improving sensitivity using the NIR channel with a sodium lamp at 0.01 lx. Figure 15a shows the result of the color restoration algorithm as an input image. Figure 15b shows that the representation of the black region is awkward, due to inaccurate linear coefficient estimates, and white line artifacts occur at strong edges. In Figure 15c, the effect of noise is greatly reduced by NIR, but a color reproduction error occurs. Figure 15d shows that color reproduction is accurate and there are no artifacts in guided filtering, but it is not enough to follow the noise characteristics of NIR. Finally, Figure 15e shows that the proposed algorithm has excellent color reproduction, spatial resolution, and noise characteristics. This is similar to the comparison of results of incandescent lamps.

## 5. Conclusions

We used an RGB-NIR MFA to replace the existing Bayer CFA. However, this method has limitations in reflecting the sensitivity of the NIR channel through the demosaic and color restoration algorithms. To solve this problem, post-processing is proposed, and an algorithm is developed to reflect three characteristics: SNR and color noise suppression at the NIR channel level, improved resolution at the NIR channel level, and RGB color image level color reproduction. For the three characteristics, we propose an edge preserving smoother that is most suitable under extremely low light conditions, by pre-processing a guided filter and estimate the linear coefficient more accurately. The edge preserving smoother helps detect the quality degradation component of the RGB-NIR MFA, such as the noise in the RGB channels. Moreover, a CBF kernel estimated from the NIR channel is applied to the U and V channels to remove color noise. The residual information is compensated by post-processing the guided filter. The proposed algorithm can estimate and compensate for the missing texture information of the guided filter result and the information of the erroneously reconstructed strong edge regions from the NIR channel. The proposed method can estimate the residual information lost in the guided filtering process by reconstructing the luminance channel into an NIR channel by using a linear coefficient. The compensation of the residual information helps improve the resolution of the output image and correct the artifacts in the strong edge areas. The experimental results show that the proposed algorithm results in a resolution and SNR similar to that of the NIR channel, under extremely low light conditions, and strictly follows the colors of the RGB channels.

## Figures and Tables

**Table 1:** Characteristics of red-green-blue (RGB) and near infrared (NIR) channels. (o means include, x means not include).

Characteristics	RGB	NIR
Sensitivity and brightness	x	o
Noise	x	o
Resolution	x	o
Local contrast for color	o	x
Hue & saturation	o	x

**Table 2:** Comparisons of signal-to-noise ratio (SNR) under extremely low light condition (0.01 lx).

0.01lx	Input RGB	Input NIR	CM1 [29]	CM2 [28]	CM3 [30]	PM
R	14.58	26.46	25.92	26.41	26.41	**27.21**
G	13.00	-	25.91	**27.00**	26.42	26.45
B	13.69	-	25.98	**26.79**	26.43	26.71
Ave.	13.76	26.46	25.94	26.73	26.42	**26.79**

## References

[1] Pohl C., Genderen J.L.V. (1998). Review article Multisensor image fusion in remote sensing: Concepts, methods and applications. Int. J. Remote Sens..

[2] Choi J., Yu K., Kim Y. (2011). A New Adaptive Component-Substitution-Based Satellite Image Fusion by Using Partial Replacement. IEEE Trans. Geosci. Remote Sens..

[3] Hao X., Chen H., Yao C., Yang N., Bi H., Wang C. A near-infrared imaging method for capturing the interior of a vehicle through windshield. Proceedings of the 2010 IEEE Southwest Symposium on Image Analysis & Interpretation (SSIAI).

[4] Hertel D., Marechal H., Tefera D.A., Fan W., Hicks R. A low-cost VIS-NIR true color night vision video system based on a wide dynamic range CMOS imager. Proceedings of the 2009 IEEE Intelligent Vehicles Symposium.

[5] Kumar A., Prathyusha K. (2009). Personal Authentication Using Hand Vein Triangulation and Knuckle Shape. IEEE Trans. Image Process..

[6] Yi D., Liu R., Chu R., Lei Z., Li S.Z. Face Matching Between Near Infrared and Visible Light Images. Proceedings of the International Conference on Biometrics.

[7] Li S.Z., Chu R., Liao S., Zhang L. (2007). Illumination invariant face recognition using near-infrared images. IEEE Trans. Pattern Anal. Mach. Intell..

[8] Salamati N., Fredembach C., Süsstrunk S. (2009). Material classification using color and NIR images. Color and Imaging Conference.

[9] Fredembach C., Susstrunk S. (2009). Illuminant estimation and detection using near-infrared. Digital Photography V.

[10] Schaul L., Fredembach C., Susstrunk S. Color image dehazing using the near-infrared. Proceedings of the 2009 16th IEEE International Conference on Image Processing (ICIP).

[11] Bayer B.E. (1976). Color Imaging Array. U.S. Patent.

[12] Bennett E.P., Mason J.L., Mcmillan L. (2007). Multispectral Bilateral Video Fusion. IEEE Trans. Image Process..

[13] Feng C., Zhuo S., Zhang X., Shen L., Susstrunk S. Near-infrared guided color image dehazing. Proceedings of the 2013 IEEE International Conference on Image Processing.

[14] Krishnan D., Fergus R. (2009). Dark flash photography. ACM Trans. Graph..

[15] Wu C., Samadani R., Gunawardane P. Same frame rate IR to enhance visible video conference lighting. Proceedings of the 2011 18th IEEE International Conference on Image Processing.

[16] Yan Q., Shen X., Xu L., Zhuo S., Zhang X., Shen L., Jia J. Cross-Field Joint Image Restoration via Scale Map. Proceedings of the IEEE International Conference on Computer Vision (ICCV).

[17] Zhang X., Sim T., Miao X. Enhancing photographs with Near Infra-Red images. Proceedings of the 2008 IEEE Conference on Computer Vision and Pattern Recognition.

[18] Zhuo S., Zhang X., Miao X., Sim T. Enhancing low light images using near infrared flash images. Proceedings of the 2010 IEEE International Conference on Image.

[19] Matsui S., Okabe T., Shimano M., Sato Y. (2010). Image Enhancement of Low-light Scenes with Near-infrared Flash Images. IPSJ Trans. Comput. Vis. Appl..

[20] Tian Q., Lansel S., Farrell J.E., Wandell B.A. (2014). Automating the design of image processing pipelines for novel color filter arrays: Local, linear, learned (L3) method. Digital Photography X.

[21] Condat L. A generic variational approach for demosaicking from an arbitrary color filter array. Proceedings of the 2009 16th IEEE International Conference on Image Processing (ICIP).

[22] Gu J., Wolfe P.J., Hirakawa K. Filterbank-based universal demosaicking. Proceedings of the 2010 IEEE International Conference on Image Processing.

[23] Park S.W., Kang M.G. (2014). Generalized color interpolation scheme based on intermediate quincuncial pattern. J. Electron. Imaging.

[24] Petrovic V., Xydeas C. (2004). Gradient-Based Multiresolution Image Fusion. IEEE Trans. Image Process..

[25] Eisemann E., Durand F. (2004). Flash photography enhancement via intrinsic relighting. ACM Trans. Graph..

[26] Petschnigg G., Szeliski R., Agrawala M., Cohen M., Hoppe H., Toyama K. (2004). Digital photography with flash and no-flash image pairs. ACM Trans. Graph..

[27] Park C., Kang M. (2016). Color Restoration of RGBN Multispectral Filter Array Sensor Images Based on Spectral Decomposition. Sensors.

[28] Li S., Kang X., Fang L., Hu J., Yin H. (2017). Pixel-level image fusion: A survey of the state of the art. Inf. Fusion.

[29] He K., Sun J., Tang X. (2013). Guided image filtering. IEEE Trans. Pattern Anal. Mach. Intell..

[30] Monno Y., Teranaka H., Yoshizaki K., Tanaka M., Okutomi M. (2019). Single-Sensor RGB-NIR Imaging: High-Quality System Design and Prototype Implementation. IEEE Sens. J..

[31] Sugimura D., Mikami T., Yamashita H., Hamamoto T. (2015). Enhancing Color Images of Extremely Low Light Scenes Based on RGB/NIR Images Acquisition With Different Exposure Times. IEEE Trans. Image Process..

[32] Montgomery D.C., Peck E.A., Vining G.G. (2012). Simple linear regression. Introduction to Linear Regression Analysis.

[33] Devi M.S., Sukumar R. (2016). Metaheuristic Based Noise Identification and Image Denoising Using Adaptive Block Selection Based Filtering. Circuits Syst..

